# The placenta of *Physcomitrium patens*: transfer cell wall polymers compared across the three bryophyte groups.

**DOI:** 10.3390/d13080378

**Published:** 2021-08-15

**Authors:** Jason S. Henry, Karen S. Renzaglia

**Affiliations:** 1 Department of Biology, One University Plaza, MS 6200, Southeast Missouri State University, Cape Girardeau, MO 63701, USA; 2 Department of Plant Biology, MC: 6509, Southern Illinois University Carbondale, Carbondale, IL 62901, USA

**Keywords:** arabinogalactan protein, cell wall, pectin, hemicellulose, transfer cell, wall ingrowth, *Physcomitrium patens*

## Abstract

Following similar studies of cell wall constituents in the placenta of *Phaeoceros* and *Marchantia*, we conducted immunogold labeling TEM studies of *Physcomitrium patens* to determine the composition of cell wall polymers in transfer cells on both sides of the placenta. 16 monoclonal antibodies were used to localize cell wall epitopes in the basal walls and wall ingrowths in this moss. In general, placental transfer cell walls of *P. patens* contain fewer pectins and far fewer AGPs than those of the hornwort and liverwort. *P. patens* also lacks the differential labeling that is pronounced between generations in the other bryophytes. In contrast, transfer cell walls on either side of the placenta of *P. patens* are relatively similar in composition with slight variation in HG pectins. Compositional similarities between wall ingrowths and primary cell walls in *P. patens* suggest that wall ingrowths may simply be extensions of the primary cell wall. Considerable variability in occurrence, abundance, and types of polymers among the three bryophytes and between the two generations suggests that similarity in function and morphology of cell walls does not require a common cell wall composition. We propose that the specific developmental and life history traits of these plants may provide even more important clues in understanding the basis for these differences. This study significantly builds on our knowledge of cell wall composition in bryophytes in general and transfer cells across plants.

## 1. Introduction

Because the sporophyte of bryophytes is matrotrophic, the placenta is the principal site for nutrient uptake that drives the production and dispersal of spores [1,2]. In this intergenerational zone, specialized cells facilitate an intensified unidirectional flow of solutes to the sporophyte that is dependent on the persistent gametophyte [3, 4]. Transfer cells characterized by localized cell wall ingrowths are common in both generations in bryophytes, but they are not universal as they may be absent or restricted to either side of the placental junction [1,5]. In transfer cells, wall ingrowths form an elaborate network or labyrinths that vastly increases the surface area of the plasmalemma, which enhances membrane-mediated nutrient transport in strategically located and specialized cell-cell junctions [3,6,4]. Wall ingrowths create a more extensive and presumably specialized apoplast and a cell wall/plasma membrane complex that is polarized and produces a directional apoplastic/symplastic exchange of solutes [7]. In addition to bryophyte placentae, transfer cells are common in tracheophytes in areas of high solute transport such as in phloem, vascular parenchyma [8], angiosperm embryos [ 9,10,11,7,12,], secretory glands [13], and root nodules [14,15]. In the placenta of mosses, carbon in the form of sucrose moves within the gametophyte symplastically and is actively loaded from the apoplast into the foot of the sporophyte [16,17]. In *Physcomitrium patens*, as in most mosses and many liverworts, transfer cells with cell wall ingrowths are located on both sides of the placenta [1]. Cell wall ingrowths and an abundance of mitochondria and plastids reflect the energy-intensive process of transferring nutrients across the extensive surface area and the dependence on proximally located sources of ATP [16]. Although the transport pathway at the placental interface of *P. patens* is beginning to be understood, little is known about the composition of wall polymers in these unique cells in mosses.

This study aims to fill in gaps in our knowledge concerning the polymer composition in bryophytes by examining the placenta of *P. patens.* Similar studies of placental cell walls in the liverwort *Marchantia polymorpha* [18] and two species of the hornwort *Phaeoceros* [5] allow for comparisons across all three bryophyte groups and across the two generations. *Phaeoceros* has transfer cells restricted to the gametophyte side, while *M. polymorpha* is similar to *P. patens* in the occurrence of transfer cells on both sides of the placenta. The placentae in *Phaeoceros* and *M. polymorpha* have AGPs not found in other parts of the plant that support signaling functions in this region of transport. In *Marchantia,* cell wall ingrowths are rich in pectins, but arabinogalactan proteins (AGPs) and xyloglucans are abundant only on the sporophyte side. In *Phaeoceros*, pectins are diverse and abundant, while AGPs are restricted to the placenta region only.

Two fundamental questions were addressed in this study: 1) How do cell wall constituents differ in the two generations of the bryophyte placenta within *P. patens?* and 2) What differences are there in composition between these cell walls and those in transfer cells of other plant groups? This comparative approach provides insights into the diversity and evolution in cell wall composition of transfer cells among the three bryophyte groups and across land plants.

## 2. Materials and Methods

### 2.1. Gametophyte culture

Mature capsules were sterilized using a 10% bleach solution. After three rinses in autoclaved distilled water, capsules were ruptured, and the released spores were sown on agar with Parker-Thompson nutrient medium. Following gametophore development, plants were transferred to vermiculite and kept in the growth chamber until antheridia and archegonia were present. Cultures were then flooded to facilitate fertilization. Plants with green capsules were harvested and processed as follows.

### 2.2. Preparation for transmission electron microscopy

For TEM observation, plants were processed according to the standard fixation protocol outlined in Renzaglia et al. 2017 [19]. Excised potions of gametophytic tissue with embedded sporophytes were fixed in 2.5% glutaraldehyde in 0.05 M Sorenson’s buffer (pH 7.2) for one hour at room temperature and overnight at 4 °C. Following 3 rinses in the same buffer for 15 min each, plants were post-fixed in 2% buffered osmium tetroxide for 15 minutes and rinsed in autoclaved, distilled water. The specimens were dehydrated in progressively higher ethanol to water concentrations and rinsed twice in 100% ethanol. Infiltration was achieved by progressively increasing the concentration of LR White resin diluted with ethanol from 25%−50%−75% and finally 100%. Specimens were exchanged three times in 100% LR White resin, placed in fresh resin in gel capsules, and cured in an oven at 60 °C for 48 h. The samples were sectioned on an ultramicrotome until the placenta was located. Either thin sections (90–100 nm) were collected on 200 mesh nickel grids for immuno-labeling, or thick sections (800 to 1500 nm) were collected on glass slides and stained with toluidine blue for light microscopy. Sporophytes with developing spores were selected and examined.

### 2.3. Immunogold labeling

The sixteen monoclonal antibodies (MAbs) in Table 1 were used to probe cell wall polymers in the placental cell walls of *Physcomitrium patens*. Specimens were processed as follows and outlined in Lopez et al. 2017 [20]. Grids were placed in BSA/PBS overnight at 14 °C, and then overnight on a primary antibody specific to the desired wall epitope. Samples were then rinsed four times in 0.05 M BSA/PBS for 4 min each. Samples were incubated overnight at 4 °C in a secondary antibody with a 10nm gold tag that attaches to the primary antibody. Samples were then rinsed in PBS four times at 4 min each and rinsed with a jet of sterile H_2_O. The primary antibody, secondary antibody, and gold tag complex attach to the desired wall epitope making the targeted wall polymer visible as a black dot in the TEM at 7000 magnification or higher. Control grids were prepared by excluding the primary antibodies.

Samples were observed before and after post-staining using lead citrate and uranyl acetate. These stains allow for better contrast but may obscure the immuno-gold labels in the TEM. Samples were viewed and digital micrographs were collected in a Hitachi H7650.

### 2.4. Scoring label intensity

Micrographs were opened in the PhotoScapeX editing app. Three counting frames sized at 100 × 100 pixels were then randomly placed onto the wall in the image. The labels within each frame were then counted and recorded. This process was repeated three times per image, and, for each MAb, 10 images were counted. The average of all counts was then calculated. An average of 1 to 4 labels per frame were assigned a single plus (+). If the average was 5 to 9 labels, two pluses (++) were given. Any averages that were greater than 10 labels per frame received a triple plus (+++). A few antibodies had scores < 1 but > 0 and were assigned a plus/ minus (±).

## 3. Results

**Figure 1.**
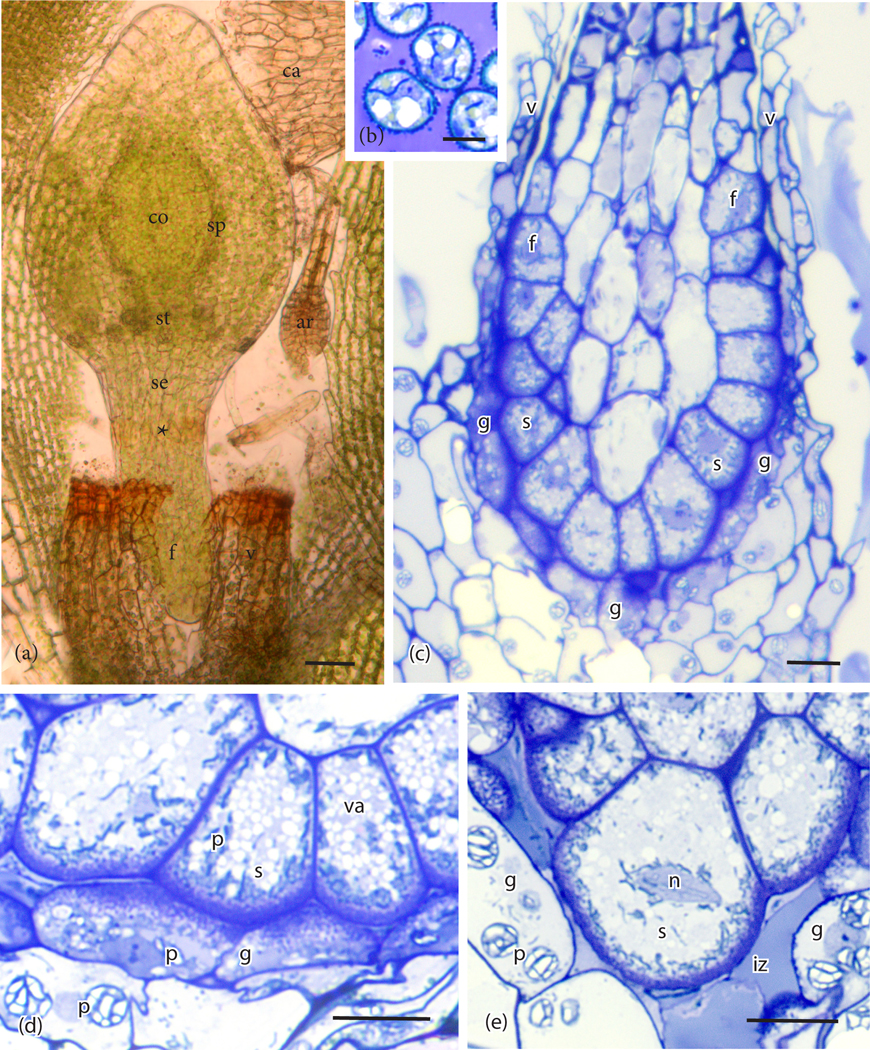
Anatomy of the sporophyte and placenta of *Physcomitrium patens.* (**a**). Developing sporophyte with expanding capsule containing a central columella (co), sporogenous layer (sp), and a zone of stomata (st) at the base. The seta (se) is short and continuous with the cylindrical foot (f) that tapers at the tip. The calyptra (ca) was dislodged from the capsule, and vaginula (v) disrupted in slide preparation to reveal the region where the foot and seta meet (*). Ar, unfertilized archegonium. (**b**). Spores in the capsule with mature placenta showing stage of development examined in (**c**), (**d**), and (**e**). (**c**). Longitudinal light microscope section of the sporophyte (s) embedded in the gametophyte (g) showing the cylindrical foot (f) at its upper limit and adjacent gametophyte transfer cells. The foot with peripheral transfer cells connects to the vaginula (v). (**d**). Along most of its length, the placenta consists of abutting sporophyte (s) and gametophyte (g) cells with extensive wall ingrowths. Sporophyte cells contain abundant small vacuoles (va) and dense plastids (p). Gametophyte cells contain rounded plastids (p) with starch grains. (**e**). At the tip of the foot, cell wall ingrowths are few, and an intergenerational zone (iz) is evident from the breakdown of gametophyte cells. Gametophyte (g) cells contain starch-filled plastids (p), and sporophyte (s) cells have numerous dense plastids (p) around the cell periphery and near the nuclei (n). *Scale bars* = 50 μm (**a**), 10 μm (**b**)-(**e**).

**Figure 2.**
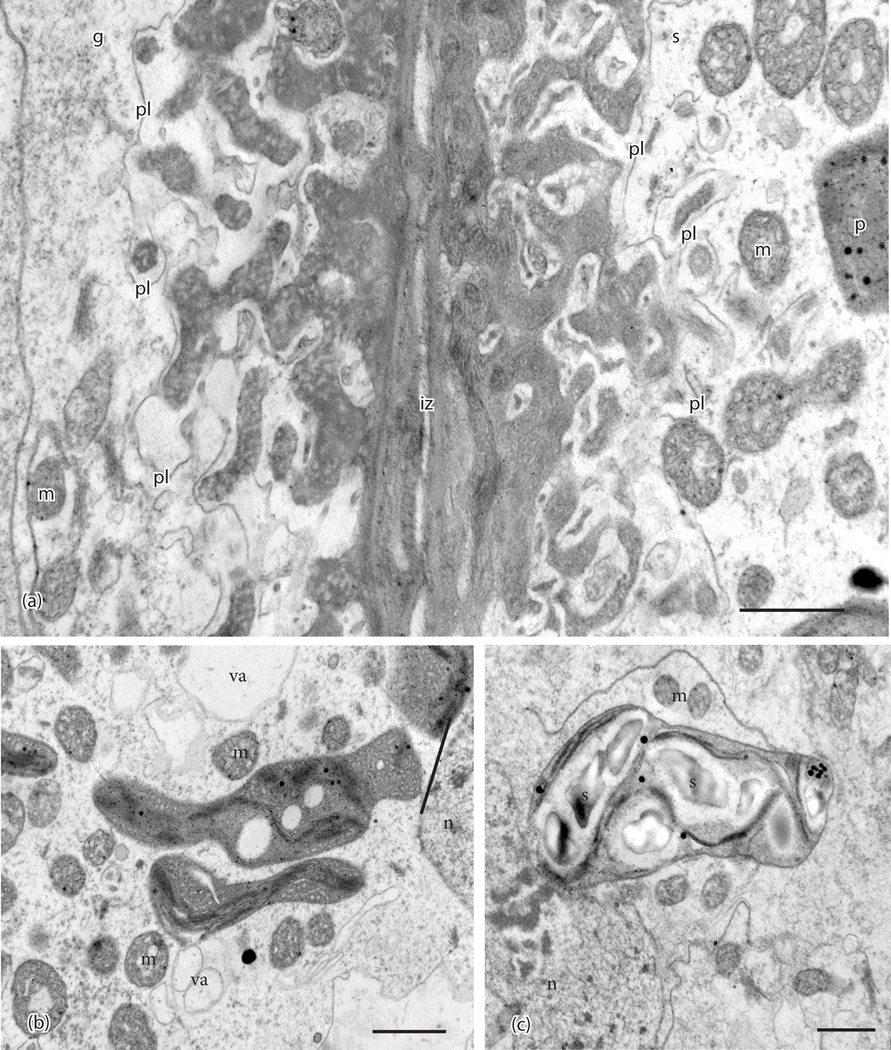
Ultrastructural details of placental cells taken in the TEM. (**a**). Transfer cells in gametophyte (g) and sporophyte (s) are separated by a narrow intergenerational zone (iz) and show elaborate cell wall labyrinths. An electron-lucent region is delimited by the plasmalemma (pl) and surrounds the dense inner core of cell wall ingrowths that is more vesicular in the gametophyte. Mitochondria (m) and plastids (p) are located near wall ingrowths. (**b**). Plastids in sporophyte (s) cells are irregular in shape, dense, vesiculate, and contain few thylakoids. Mitochondria (m) and small vacuoles (va) are numerous in sporophyte cells. n, nucleus. (**c**). Plastids in gametophyte (g) cells contain starch grains (s) surrounded by thylakoids. m, mitochondria; n, nucleus. *Scale bars =* 0.1 μm

The foot of *P. patens* is small, typically less than 500μm long, and 6 or 7 cells in diameter (Fig. 1a). A ring of gametophytic tissue (vaginula), derived from the archegonium, surrounds the foot that is cylindrical and gradually tapers to a pointed tip where it penetrates the gametophore. The vaginula ensheaths the foot along most of its length. The foot is fully developed when the sporophyte capsule begins to expand, and sporogenous tissue is delimited (Fig. 1a). At this stage, the capsule is emerging from beneath the calyptra, and stomata are developed. Transfer cells reach maturation and line both sides of the placenta by the time meiosis is completed and persist throughout spore differentiation (Figs. 1b, c). Cell wall ingrowths are generally more elaborate on the gametophyte side of the placenta compared with the foot side, and they are less abundant at the tip of the foot (Figs. 1d, e, 2a). Wall ingrowths in both gametophyte and sporophyte transfer cells contain a fibrous core (sporophyte side) or vesicular dense core (gametophyte side) and an irregular outer electron lucent zone that is bordered by plasmalemma (Fig. 2a). Along the sides of the foot, the two generations make contact and the intergenerational zone is obscured (Figs. 1c, d, 2a). At the foot tip, degenerating gametophyte cells leave a mucilaginous matrix (Fig. 1e). Transfer cells of the foot are more isodiametric than those of the gametophyte, and they contain numerous small vacuoles and peripheral cytoplasm with numerous mitochondria and elongated plastids with dense stroma, few membranes, and no starch (Figs. 1d, 2b). Gametophyte transfer cells contain dense cytoplasm with prominent rounded plastids that are rich in starch (Figs. 1d, e, 2c)

**Figure 3.**
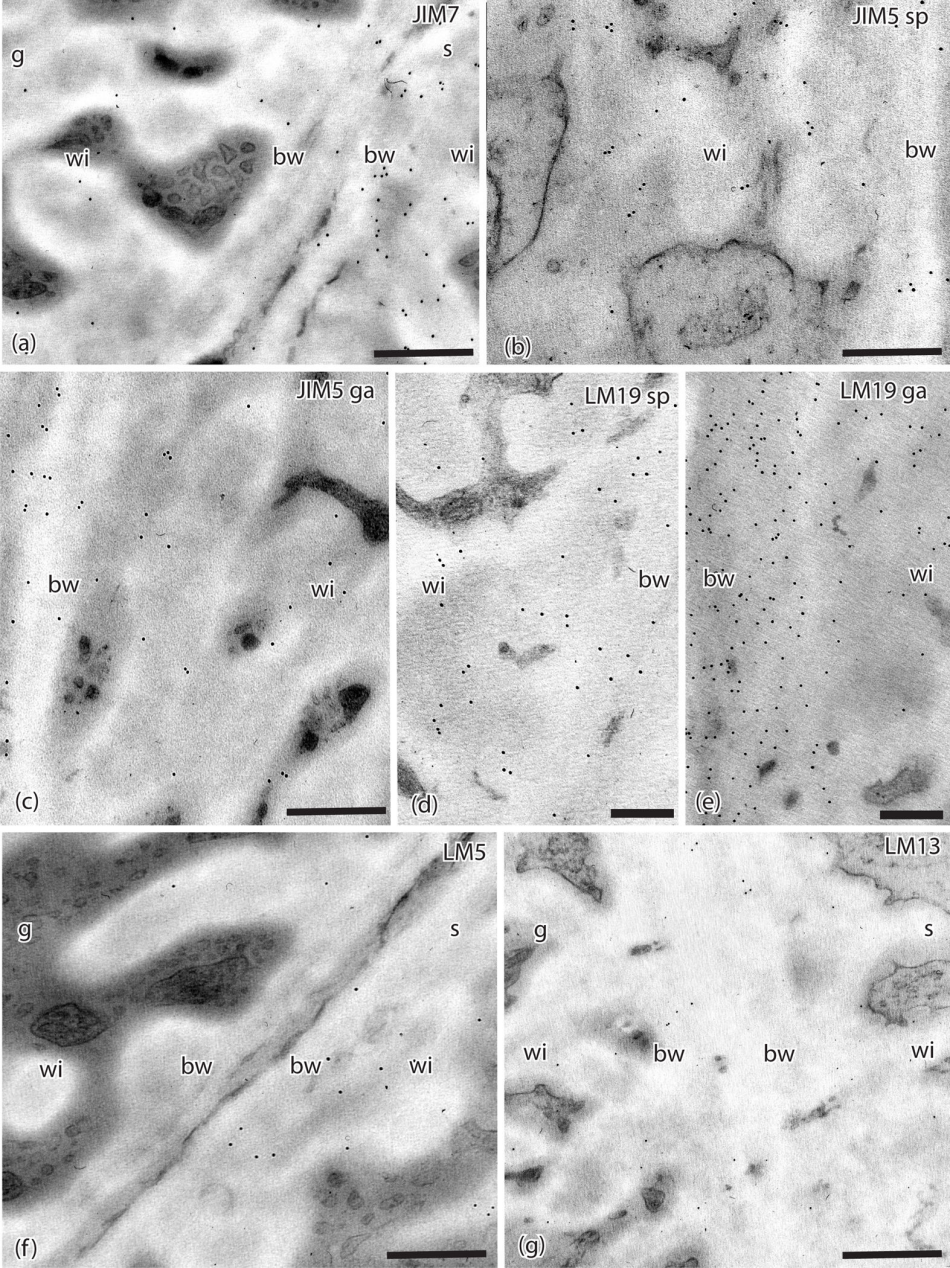
TEMs of *Physcomitrium patens* placenta. Immunogold labeling with monoclonal antibodies to pectin epitopes. (**a**). JIM7 labels sporophyte (s) placental cell walls with more abundance than those in the gametophyte (g). Labeling occurs throughout the basal wall (bw) and the wall ingrowths (wi) on the sporophyte side. (**b**). JIM5 labels the basal wall (bw) and wall ingrowths (wi) in sporophyte transfer cells. (**c**). JIM5 labels the basal wall (bw) and wall ingrowths (wi) in gametophyte transfer cells. (**d**). LM19 labels are found in sporophyte basal wall (bw) and wall ingrowths (wi). (**e**). LM19 labels on the gametophyte side are more abundant in both basal walls (bw) and wall ingrowths (wi) compared to the sporophyte cell walls. (**f**). LM5 and (**g**) LM13 sparsely label the basal wall (bw) and wall ingrowths (wi) in both the gametophyte (g) and sporophyte (s) placental cells. *Scale bars* = 0.5 μm.

Label intensity in the sporophyte and gametophyte cell walls for the 16 MAbs used in this study is summarized in Table 2. Three of the four MAbs for HG pectins localize epitopes of these pectins in the *P. patens* placenta (Fig. 3). Labeling with JIM7 for methyl-esterified HG pectins is light in the gametophyte basal wall layer and wall ingrowths (Fig. 3a). Moderate labeling for this MAb occurs in sporophyte transfer cell walls (Fig. 3a). The JIM5 MAb that also targets a de-esterified HG epitope shows light labeling in electron dense regions in both the basal wall layer and cell wall ingrowths in both generations (Figs. 3 b, c). The LM19 MAb recognizes de-esterified HG labels throughout the electron dense portions of the cell wall ingrowths as well as the basal wall in both generations (Fig. 3 d, e). No labeling was observed for the LM20 MAb.

The presence of RG-I pectins was identified by two MAbs (Tables 1 and 2). The LM5 MAb lightly labels the basal wall layer and wall ingrowths on the sporophyte side and less so on the gametophyte side (Fig. 3f). The LM13 MAb shows very light labeling in the basal cell wall and wall ingrowths in both generations (Fig. 3g).

**Figure 4.**
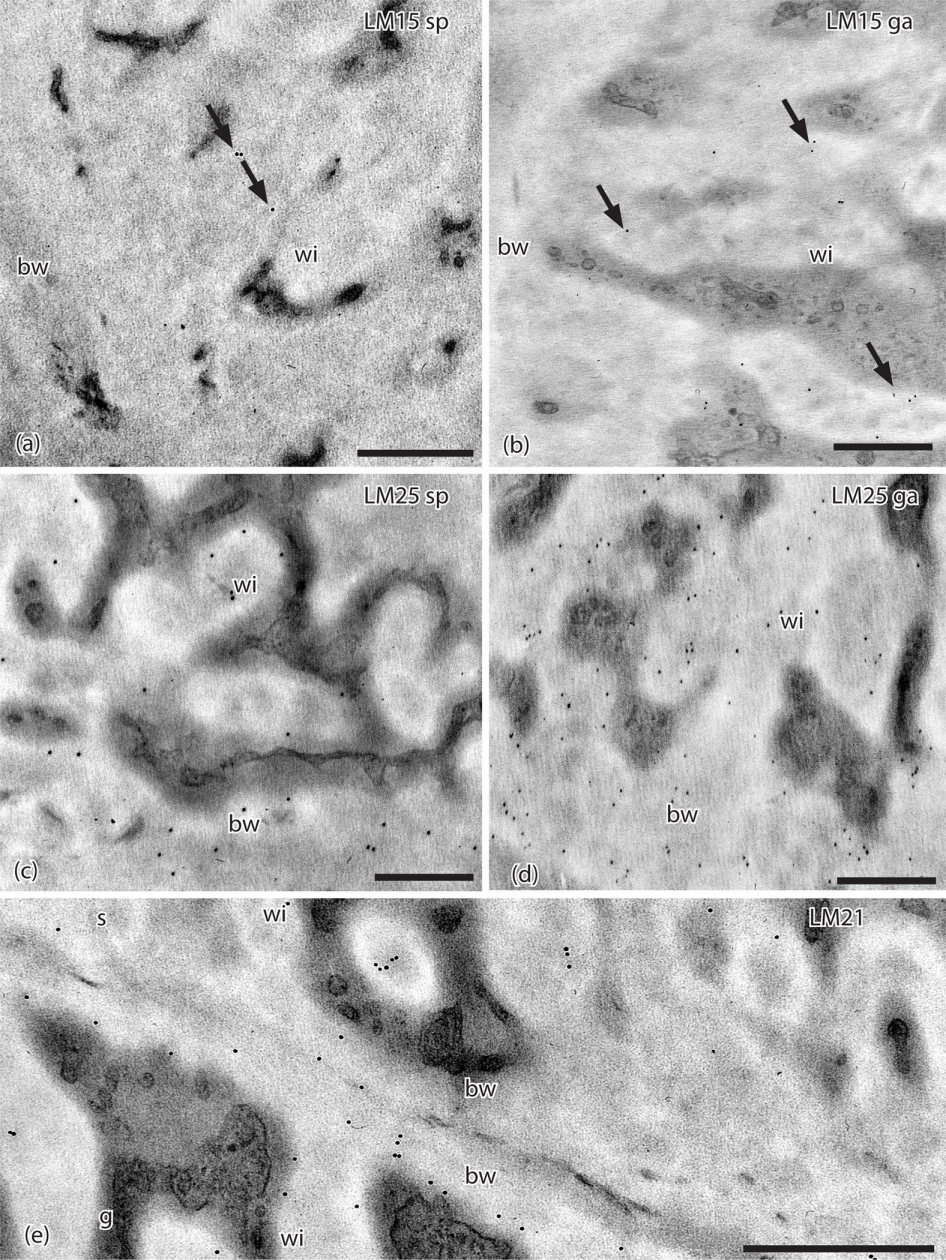
TEMs of *Physcomitrium patens* placenta. Immunogold labeling with monoclonal antibodies to hemicellulose epitopes. (**a**). LM15 does not label the basal wall (bw) and sparsely labels (arrows) sporophyte cell wall ingrowths (wi). (**b**) LM15 does not label the basal wall (bw) and sparsely labels (arrows) gametophyte cell wall ingrowths (wi). (**c**). LM25 labels sporophyte placental cell wall ingrowths (wi) and the basal wall (bw). (**d**). LM25 labels gametophyte placental cell wall ingrowths (wi) and the basal wall (bw). (**e**). LM21 labels sporophyte (s) and gametophyte (g) transfer cell wall ingrowths (wi) and basal walls (bw). *Scale bars* = 0.5 μm.

The LM15 hemicellulose MAb targeting xyloglucan sparsely labels cell wall ingrowths in both generations (Figs. 4 a, b, Table 2). Galactoxylated xyloglucans as localized with the LM25 MAb are found in the basal wall and wall ingrowths in transfer cells in both generations with fewer labels on sporophyte walls (Figs. 4c, d). The LM21 MAb that is specific to mannans lightly labels both generations in electron dense areas near the basal wall layer and in wall ingrowths near the plasmalemma (Fig. 4e). No labels were detected for the LM28 MAb.

**Figure 5.**
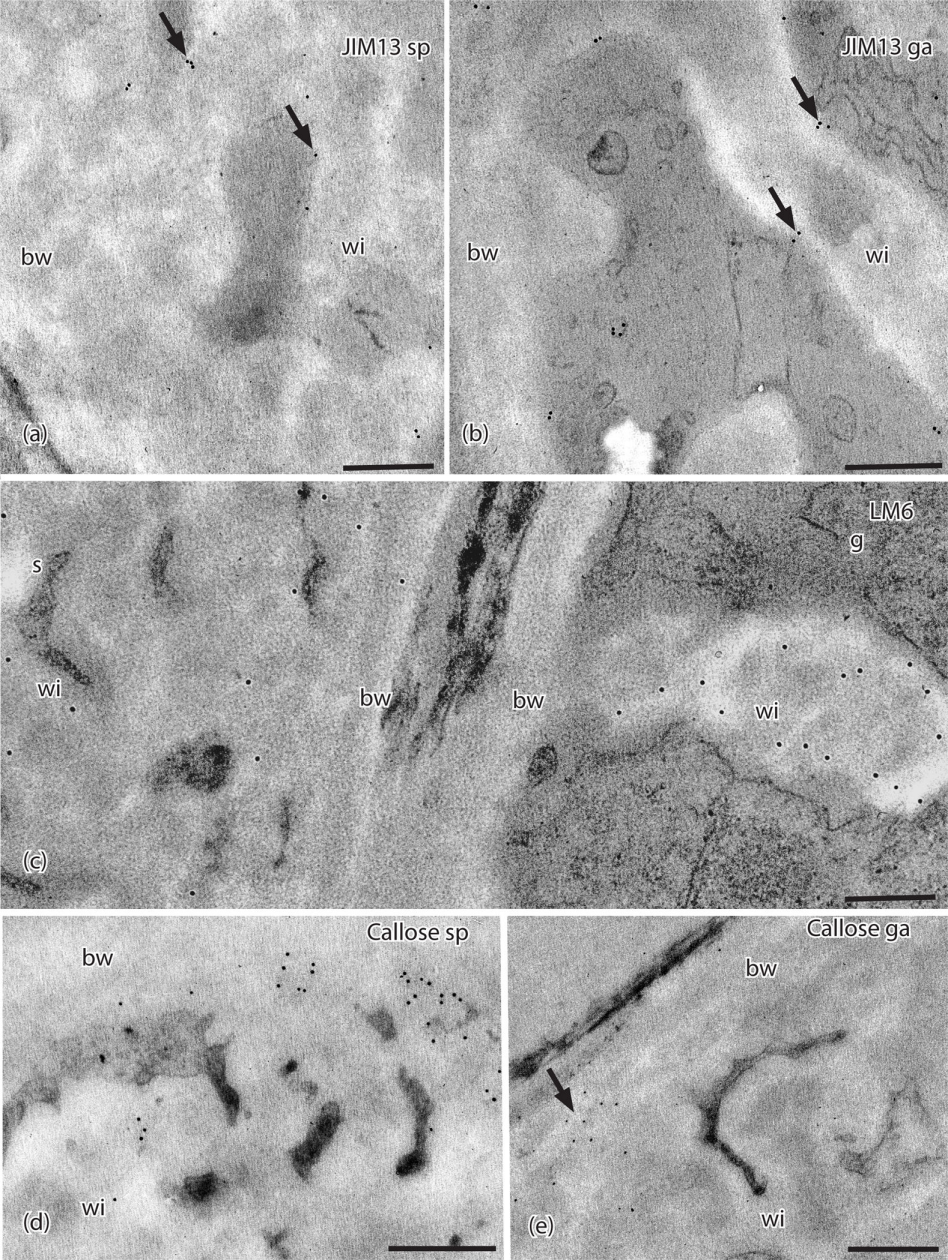
TEMs of *Physcomitrium patens* placenta. Immunogold labeling with monoclonal antibodies to AGP and callose epitopes. (**a**) In the sporophyte placental transfer cell, JIM13 labels (arrows) occur along the plasma membrane and wall ingrowths (wi) but not in the basal wall layer (bw). (**b**) Labels for JIM13 (arrows) occur in the gametophyte along the plasma membrane and wall ingrowths (wi) but not in the basal wall (bw). (**c**) LM6 labels are scattered throughout the wall ingrowths (wi) and basal wall (bw) in the sporophyte (s) and mostly in the electron lucent area along the edges of the wall ingrowths (wi) in the gametophyte (g) side, with few labels in the basal wall (bw). (**d**) Sporophyte and (**e**) Gametophyte. Labels for anti-callose (arrows) appear along the outer edge of the basal wall (bw) where it comes into contact with the wall ingrowths with few labels in wall ingrowths (wi). *Scale bars =* 0.5 μm.

Of the four AGP-targeting MAbs, only JIM13 and LM6 localized in the placenta of *P. patens* (Tables 1 and 2). JIM13 epitopes are found along the plasmalemma in wall ingrowths in both generations (Figs. 5a, b). Labels for the LM6 MAb targeting AGPs are scattered along electron lucent regions of wall ingrowths in both generations with few labels in the basal wall layer (Fig. 5c). The JIM8 and LM2 MAbs do not label placental cell walls in *P. patens*.

Callose, as labeled with the anti-callose MAb, occurs in the sporophyte placental transfer cells in electron dense area where the basal wall layer transitions to wall ingrowths (Fig. 5d, Table 2). Light labeling of anti-callose is seen in clusters throughout the basal wall of gametophyte placental cells (Fig. 5e). Extensin, as labeled with the JIM12 MAb, was not detected in *P. patens* placental cell walls.

## 4. Discussion

The cylindrical foot of *P. patens* extends only slightly into the gametophyte tissue, forming a tapering extension of the short seta. Because the sporophyte takes approximately one month to reach maturation after fertilization, the placenta is short-lived compared to most mosses in which the sporophyte is long-lived, typically one year [74]. Interestingly, the placenta of *M. polymorpha* is similarly short-lived as the sporophyte also completes development in approximately one month. The placenta of both *P. patens* and *M. polymorpha* contains transfer cells with elaborate wall labyrinths on both the sporophyte and gametophyte sides. The massive bulbous foot of *Phaeoceros*, in turn, persists through the growing season, over many months, placing a continuous demand on the gametophyte for nutrient transport across generations. The foot side of the placenta in this hornwort is lined in elongated cells that lack wall ingrowths. During development, these haustorial cells penetrate and interdigitate with gametophytic cells that contain extensive wall ingrowths. These anatomical and developmental differences may account in part for the considerable variability in occurrence, abundance, and types of polymers across the placental cells of these three bryophyte taxa and between the two generations.

**Figure 6.**
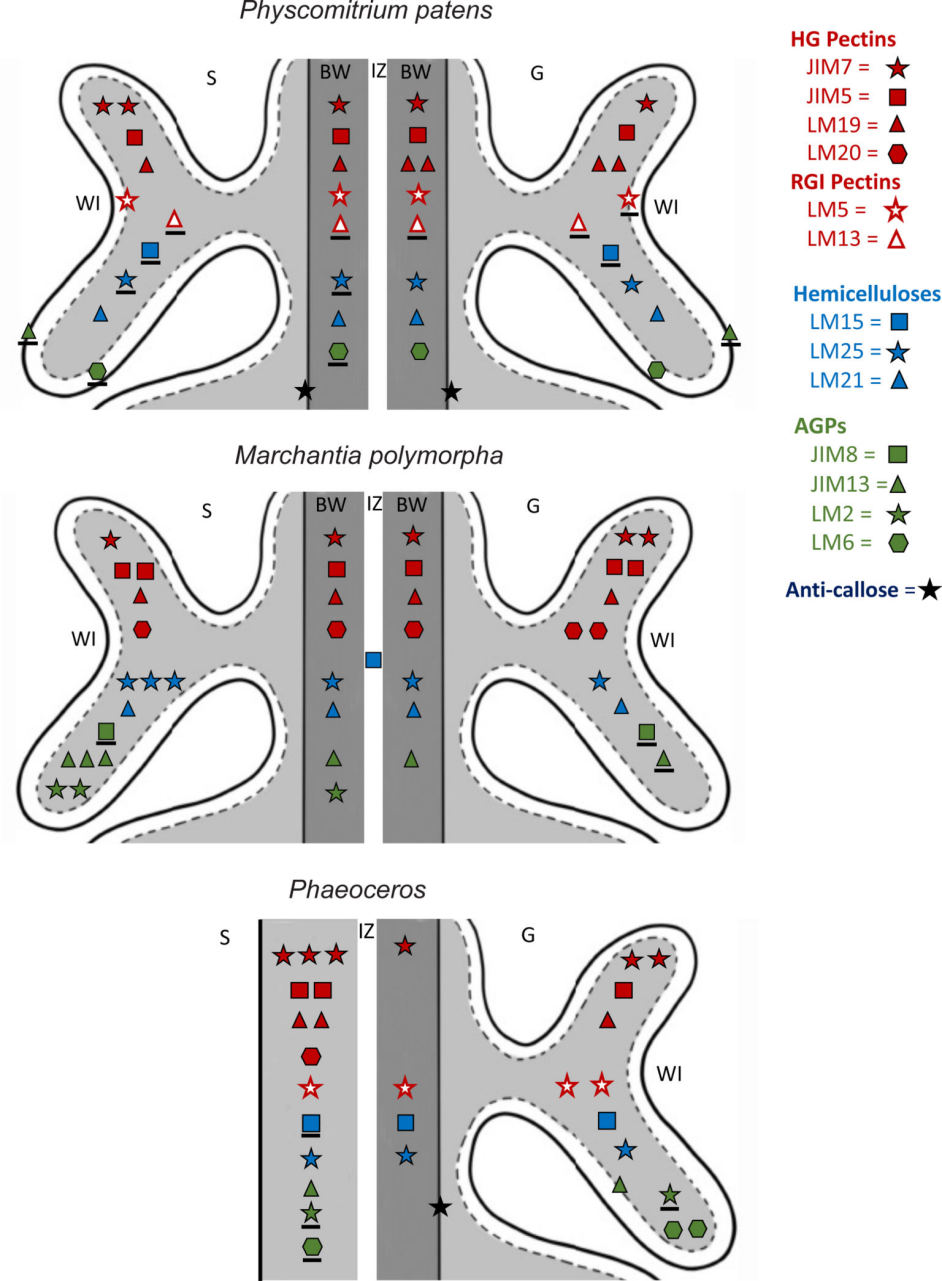
Comparative diagrammatic representation of labeling with 14 MAbs to epitopes of cell wall polymers in three bryophyte placentae: the moss *Physcomitrium patens* (this study), liverwort *Marchantia polymorpha* [18], and two species of *Phaeoceros* [5]. The LM28 and LM12 MAbs yielded no labels in any plant. LM13 labeling in *Phaeoceros* was inconclusive and omitted. Pectins, red; hemicellulose, blue; AGPs, green; callose, black. The number of symbols per MAb corresponds to label intensity as follows: three symbols, very strong; two symbols, strong; one symbol, weak; underlined symbol, present but rare.

As in other bryophytes, cell wall constituents in the *P. patens* placenta include diverse polymers that include pectins, hemicelluloses, AGPs, and callose (Table 2). In general, placental transfer cell walls of *P. patens* contain fewer pectins and far fewer AGPs than those of *M. polymorpha* and *Phaeoceros* (Fig. 6). Transfer cell walls on either side of the placenta of *P. patens* are relatively similar in composition with slight variation in HG pectins. In the other two bryophytes, cell walls are more variable in abundance and type of polymers across generations, which is especially evident in *M. polymorpha* (Fig. 6).

Pectins are GalA-containing polysaccharides that often account for a large portion (~30%) of the primary cell wall of most angiosperms [75,76,77]. The pectin composition imparts porosity, permeability, and flexibility to primary walls [78] (Table 3), cell wall properties important to the development and directional transport of placental walls [18]. In the placenta of *P. patens,* pectins are diverse in both generations (Fig. 6). Pectins show variable distribution and are particularly abundant on the sporophyte side of the *Phaeoceros* placenta (Fig. 6), which is likely related to the unique intrusive growth of the foot cells and the requirement of haustorial cells to elongate unidirectionally [5].

HG pectins play significant roles in cell wall properties and mechanics, and thus affect functions such as apoplastic transport [78] (Table 3). HG is laid down in the methyl-esterified form, which is stretchable, porous, lower apoplastic pH which facilitates nutrient uptake by membrane transport proteins [79]. These properties are consistent with the function of transport in placental transfer cell walls, which explains the widespread occurrence of JIM7 epitopes in these bryophytes (Table 3, Fig. 6). The LM20 MAb that also recognizes methyl-esterified pectins was not detected in *P. patens,* but epitopes of this MAb occur in both generations in *Marchantia* and on the sporophyte side only in *Phaeoceros*. LM20 epitopes are found in other bryophyte tissues, including the developing gametophore apex and rhizoids of *P. patens* [80, 68]. The more rigid un-esterfied HG epitopes (LM19, LM5 MAbs) are common across both generations in the three bryophytes, with higher abundance in the gametophyte than sporophyte cell walls in *P. patens*, and the reverse in *Phaeoceros* (Fig. 6). Methyl-esterified HGs also occur in the wall ingrowths on both sides of the placenta in *Ceratopteris* [81], in epidermal transfer cell walls of *Vicia* [82], and the basal wall layers (but not wall ingrowths) in transfer cells of *Elodea* [83]. In *Elodea,* un-esterfied HG epitopes were not detected in transfer cell wall ingrowths but do occur in other wall layers [83].

In contrast to HG pectins that are long-chain polymers, RG-I and RG-II have complex side-chain configurations associated with them. The absence of antibodies to RG-II pectins and the low levels of their occurrence in bryophytes (estimated to 1% of the amount in angiosperm cell walls [84]) has limited our understanding of where they occur in bryophytes. MAbs that detect RG-I pectin, both (1–5)-α-L-arabinans (LM13) and (1–4)-β-D-galactans (LM5)-containing, show relatively low levels of labeling in both generations in *P. patens*. In *Phaeoceros*, the highest level of labeling of LM5 was present in the gametophyte transfer cell walls, while in *P. patens* in the sporophyte transfer cell walls. The placenta of *M. polymorpha* does not label with MAbs (LM5, LM13) for RGI pectins (Fig. 6) [18]. Galactan-rich RG-I is also present in the epidermal transfer cell walls of *Vicia*, which sparsely contain LM5 epitopes [82]. However, this MAb does not label transfer cell walls in *Ceratopteris* or *Elodea* [81,83]. The presence of galactan-rich RG-I pectin domains in moss and hornwort placentae is consistent with the hypothesized role of these pectins in directional growth as in root epidermal cells of *Arabidopsis* seedlings, where they are thought to act as molecular markers for the cell elongation transition zone [85,40]. Although RG-I pectins are not abundant in the primary cell walls of bryophytes and ferns [25,49], labeling for the LM5 MAb has been observed in the water-conducting cells in some mosses and liverworts [80,86]. These polymers have also been observed in small amounts in *P. patens* rhizoids [68] and protonemal cells, and in the rhizoids of *Ceratopteris* [87].

Xyloglucans (LM15), galactoxyloglucan (LM25), and mannans (LM21) are hemicellulose constituents of the placental cell wall in *P. patens* (Table 2). As in *Marchantia* and *Phaeoceros*, there is no labeling for glucoronoxylans (LM28) in either generation. Galactoxyloglucan is more abundant than xyloglucans in the *P. patens* placenta, especially on the gametophyte side. In *Phaeoceros,* light labeling for galactoxyloglucan occurs in both generations, and in *Marchantia*, these epitopes are more abundant in the sporophyte generation. Using a polyclonal antibody for xyloglucan, Vaughn et al. [82] observed an abundance of this polymer in *Vicia* transfer cell walls. Xyloglucans are common cell wall polymers known to associate with both cellulose networks and acidic pectins across land plants [85, 88]. A possible function in transfer cell walls is as a regulator of cell wall extensibility by weakening the cellulose network to allow slippage during cell growth (46, 43).

The transfer cell walls of both generations in *P. patens* have low levels of mannan-containing hemicellulose, which is similar to the placenta of *Marchantia* but differs from that of *Phaeoceros* that lacks mannans. Because mannans also occur in protonemata and rhizoids in *P. patens,* these polymers have been speculated to facilitate nutrient uptake, water sensing, and cell wall reinforcement, all of which would be important and useful properties for transfer cell walls (Table 3) [49,50,89]. The combination of mannans and small amounts of arabinan-containing RG-I pectin in *P. patens* may enhance water and nutrient movement while the small amounts of galactan-containing RG-I may increase rigidity of these walls [49,50,89].

AGPs are proteoglycans made of a protein backbone that is heavily O-glycosylated (90% of the overall mass). As seen in Figure 6, arabinogalactan proteins (AGPs) are the most variable cell wall polymers present in bryophyte placental cell walls. Transfer cell walls in the *P. patens* placenta have lower diversity and amounts of AGPs than in *Marchantia* and *Phaeoceros.* Gametophyte cell walls in the placenta are only slightly richer in AGPs than those of the sporophyte in *P. patens.* In *Marchantia,* sporophyte cell wall ingrowths show an abundance of AGPs compared with gametophyte wall ingrowths. In gametophyte transfer cells, AGP labeling is light in *Phaeoceros* and even less abundant in *Marchantia*. AGPs are abundant in placental transfer cell walls of the *Ceratopteris* gametophyte with less labeling observed in sporophyte cells [81]. Vaughn et al. (2007) [82] found AGP epitopes in *Vicia* wall ingrowths along the plasmalemma around the outer edges of cell wall ingrowths adjacent to an electron-lucent layer that contains callose. AGP epitopes were not detected in *Eldoea* leaf transfer cells [83].

The varied and important roles of AGPs in plant biological processes are increasingly becoming clear [90]. These proteoglycans are speculated to be involved in differentiation, cell to cell recognition, embryogenesis, programmed cell death, tip-growth, pectin plasticization, and pH-dependent signaling by releasing Ca2+ as a secondary messenger that regulates development [91,92,93,94,52] (Table 3). The contribution of AGPs to placental development and functions is likely varied. Regulated signaling by AGPs would facilitate the interaction between generations, and the systematic directional transport of nutrients. In angiosperms, cytosolic Ca2+ accumulation is important in the development of reticulate cell wall ingrowths that are similar to those in bryophytes [95]. AGPs are also hypothesized to act as markers that aid in directing the polarized growth of wall ingrowths [96]. AGPs may act as pectin plasticizers by preventing HG domain crosslinking [97].

Across bryophytes, AGPs have been observed in the walls of water-conducting cells in both mosses and liverworts [86], in apical cell extension of protonemata and water balance in *P. patens* [54,98] and in hyaline cell walls in *Sphagnum* [99]. AGPs have been implicated in protonemata differentiation [100], cell wall regeneration of the cultured protoplasts [101], and cell plate formation in *M. polymorpha* [102]. Their significance in sexual reproduction has been observed in the process of spermatogenesis in *Ceratopteris* [46] and the moss, *Aulacomnium palsutre* [103]. The female gametes of *Ceratopteris* also express AGPs during development [55].

Callose occurs in both generations of *P. patens* along the electron-dense base of wall ingrowths. Callose labeling does not occur in the *Marchantia* placenta, and in *Phaeoceros* it is restricted to the gametophyte generation around plasmodesmata. In contrast, callose is more prominent in *Vicia* and *P. sativum* cell wall callose where it is localized in the electron-dense areas of wall ingrowths and basal wall [83,104].

As has been observed in tracheophytes, unique cell wall compositions characterize transfer cells across taxa, and the variability in the placentae of the three bryophytes may be explained in part by differences in the developmental and physiological interactions between the generations, and the longevity of the sporophyte and associated protective structures. It is logical to link the differential polymer composition in the placenta of *Phaeoceros* to differences in development and function between gametophyte transfer cells and sporophyte haustorial cells [5]. Because the placenta of hornworts is long-lived and nutrient demands from the growing sporophyte is high, differential cell wall composition would make sense for efficient unidirectional transport [5]. Generational differences in cell wall polymers are also evident in *Marchantia* and these also likely reflect directional movement [18]. In this liverwort, the foot is small and anchor-shaped and the sporophyte is surrounded by three protective structures (calyptra, pseudoperianth and involucre) throughout development [105,18]. A constant nutrient transport via the gametophyte would be critical for sporophyte development as photosynthetic activity of this generation is limited. Compared to most moss sporophytes that persist for approximately one year, the longevity of the *P. patens* sporophyte is highly abbreviated. The sporophyte is green throughout development with significant autonomy. This may explain the lack of generational variability in placental transfer cell walls in this moss. Although placental cell walls in *P. patens* are less pectin and AGP-rich than those of *M. polymorpha* and *Phaeoceros*, the ratios of carbohydrates in these transfer cell walls are similar to those described in moss primary cell walls [106, 88, 80]. Compositional similarities between wall ingrowths and primary cell walls suggest that wall ingrowths may simply be extensions of the primary cell wall in *P. patens* as hypothesized in *Vicia* by Vaughn et al. [82]. Whether this hypothesis is valid or *P. patens* placental cell walls simply represent an evolutionary reduction in complexity awaits further testing with immunolabelling on placentae of mosses with more extended life cycles.

Transfer cells evolved multiple times and are important in directional transport and tissue function across algae, fungi and plants [107,15,108,109]. It is therefore surprising that transfer cell wall composition is poorly characterized and known only from *Elodea*, *Vicia, Pisum* and the placenta of three bryophytes. The occurrence, abundance, and types of polymers is considerably different among these taxa and between the two generations, suggesting that similarity in function and morphology of cell walls does not require a common cell wall composition. We propose that the specific developmental and life history traits of plants may provide even more important clues in understanding the basis for these differences. Understanding what polymers are present, their abundance and their associations with each other is foundational to further work on plant cell walls. Additional studies of cell wall polymers on a broad spectrum of tissue types across bryophyte diversity are necessary to assess the variability in cell wall composition and its impact on the function and evolution of cell walls across plants.

## Figures and Tables

**Table 1.** Primary monoclonal antibodies (MAbs) used to immunogold label carbohydrates and arabinogalactan proteins in placental cell walls of *Physcomitrium patens*.

Antibody	Antigen (s)/ Epitope	Reference/ Source
JIM7	Homogalacturonan/ Methyl-esterified	21
JIM5	Homogalacturonan/ Un-esterified	22
LM19	Homogalacturonan/ Un-esterified	23
LM20	Homogalacturonan/ Methyl-esterified	23
LM5	Galactan, rhamnogalacturonan-I/(1–4)-β-d-galactan	24
LM13	Arabinan, rhamnogalacturonan-I/(1–5)-α-L-arabinan (linear)	25
LM15	XXXG motif of xyloglucan	26
LM25	Galactoxylated xyloglucans	27
LM21	Mannan/ β-(1,4)-manno-oligosaccharide	28
LM28	Glucuronoxylan	29
JIM13	Arabinogalactan protein (AGP)/β-d-GlcA-(1,3)-α-d-GalpA-(1,2)-l-Rha(glucuronicacid-galacturonicacid-rhamnose)	30
LM6	Arabinan, rhamnogalacturonan-I/(1–5)-α-L-arabinan(also labels AGP)	31
JIM8	Arabinogalactan protein (AGP)/ unknown	32
LM2	Arabinogalactan protein (AGP)/ β-d-GlcA (glucuronic acid)	33
Anticallose	Callose/ (1,3)-β-linked penta-to-hexa-glucan	34
JIM12	Extensin	35

**Table 2.** Relative intensity of immunogold labeling for sporophyte and gametophyte placental cell walls in *P. patens* with 16 monoclonal primary antibodies.

Primary Antibody	Sporophyte	Gametophyte
JIM7	++	+
JIM5	+	+
LM19	+	++
LM20	-	-
LM5	+	±
LM13	±	±
LM15	±	±
LM25	±	+
LM21	+	+
LM28	-	-
JIM13	±	±
LM6*	±	+
JIM8	-	-
LM2	-	-
Callose	+	+
JIM12	-	-

Notes:

+++, very strong

++, strong

+, weak

±, present

−, absent

* LM6 binds to arabinan residues in RG-I pectins and AGPs.

**Table 3.** Cell wall polymers, the MAbs that target them, their reported properties and the supporting references.

Cell wall polymer		MAbs	Wall properties	References
HG Pectin	Esterified	JIM7 , LM20	Porosity and permeability Expansibility Elasticity	36,37,38
	De-esterified	LM19, JIM5	Ca^2+^ binding Rigidity Resistance to mechanical stress Cell-to-cell adhesion	37,39,38
RG-I Pectin	Arabinan	LM13, LM6*	Spatial buffer Flexibility Expansibility and elasticity Porosity to wall Increases water holding capacity Signaling	37,40,41,39
	Galactan	LM5	Rigidity Tip growth	40,37
Hemicellulose	Xyloglucan	LM15, LM25	Regulates expansibility and yield threshold Cell-to-cell adhesion Cross-linkage/ tethering Nutrient source Sexual reproduction	36,42,43,44,45, 46
	Mannan	LM21	Anchoring Interacts with soil particles, microorganisms Nutrient uptake Hydrated/de-hydrated cycles Cross-links with cellulose Nutrient source	47,48,49,50
AGP		JIM13, JIM8, LM2, LM6*	Development Cell identity Structural integrity to walls Galactan turnover Ca^2^+ regulation/signal transduction Plasticity — unidirectional deformation Desiccation tolerance Membrane integrity Tip growth Sexual reproduction	51,40,52,53,46,55
Extensin		JIM12	Cell wall assembly and growth Tip growth Cell wall/cytoplasm communication	56,57,58,59
Callose		Anticallose	Stress response Sieve plate/ sieve areas Scaffolding for cell plate formation Plasmodesmata Developmental processes Tip growth/ Pollen tube Spore wall development/structure Sperm cell differentiation Desiccation tolerance	60,61,62,63,64, 65,66,67,68,69, 70,71,72,73

* LM6 detects arabinan sidechains in both AGPs and pectin.
